# Apex vs. Septum Pacing: A Comprehensive Review of Pacemaker Implantation Strategies

**DOI:** 10.3390/biomedicines13081822

**Published:** 2025-07-25

**Authors:** Yashar Jalali, Ján Števlík

**Affiliations:** 1 Faculty of Medicine, Comenius University in Bratislava, 5th Department of Internal Medicine, University Hospital Bratislava, Ružinov, Špitálska 24, 813 72 Bratislava, Slovakia, and Ružinovská 4810/6, 821 01 Bratislava, Slovakia; 2 5th Department of Internal Medicine, University Hospital Bratislava, Ružinovská 4810/6, 821 01 Bratislava, Slovakia; stevlik@ru.unb.sk

**Keywords:** cardiac pacemaker, right ventricular apex pacing, left bundle branch area pacing, his bundle pacing, pacemaker-induced cardiomyopathy

## Abstract

Right ventricular apex (RVA) pacing has historically been the default approach for cardiac pacing; however, it is associated with the development of progressive left ventricular dysfunction and heart failure (HF), particularly in patients with high pacing burdens. While advances in device programming and modern algorithms have sought to mitigate these effects, preserving physiological activation has proven to be more critical than reducing ventricular pacing. Conduction system pacing (CSP) techniques—namely, His-bundle pacing (HBP) and particularly left bundle branch area pacing (LBBAP)—have emerged as superior alternatives, enabling improved left ventricular function and reduced rates of pacing-induced cardiomyopathy (PICM). Nevertheless, despite the clinical advantages of these procedures over RVA, they face limitations including variable implantation success rates, increased pacing thresholds and lead revision rates, technical challenges, and occasional procedure prolongation. Thus, while CSP approaches represent the future of physiological pacing, RVA pacing continues to provide a necessary and reliable option in the current clinical practice.

## 1. Introduction

Permanent cardiac pacing has revolutionized the management of bradyarrhythmia since the first successful implantation of a pacemaker in the late 1950s. Over subsequent decades, technical advancements have refined both the functionality of the used devices and lead placement strategies [1]. Traditionally, the RVA was favored as the standard pacing site due to its accessibility, procedural simplicity, and reliable sensing and capture thresholds [1]. This strategy quickly became the clinical default and remains predominant in many practices.

However, accumulating evidence over the past two decades has highlighted significant drawbacks associated with chronic apical pacing. Specifically, RVA pacing induces an unnatural pattern of ventricular activation that mimics left bundle branch block (LBBB), resulting in interventricular and intraventricular desynchrony. This electrical desynchrony can lead to PICM, which increases the risk of HF hospitalization and mortality [2,3,4].

In light of these findings, interest has surged in alternative pacing sites that may offer more physiological activation of the myocardium. Right ventricular septal (RVS) pacing has emerged as a promising option, aiming to minimize electrical desynchrony by pacing closer to the intrinsic conduction system. Randomized studies—such as the SEPTAL-PM trial—have demonstrated that RVS pacing, reduces HF hospitalizations, and lowers the risk of PICM compared to RVA pacing [5].

In parallel with efforts to refine RV pacing sites, CSP—specifically HBP and LBBAP—has garnered significant attention as a direct means of restoring physiological ventricular activation. HBP realizes the theoretical ideal of “purely physiological” activation by directly engaging the His–Purkinje system. Studies have shown that HBP improves ventricular synchrony and clinical outcomes, even in direct comparison with more advanced resynchronization methods such as biventricular pacing [6]. Nevertheless, despite these theoretical and early clinical advantages, HBP has been shown to be associated with a high pacing threshold, increased lead revision rates, and technical challenges during implantation [7,8,9,10].

LBBAP, as a more recent innovation, addresses many of the technical limitations of HBP (e.g., lead instability and high pacing thresholds) and has demonstrated excellent electrical parameters, narrower QRS complexes, and superior preservation of ventricular function compared to RVA pacing [11]. However, LBBAP itself is not free from issues: concerns regarding lead depth penetration, risk of septal perforation, and difficulty in achieving consistent lead positioning persist [12,13,14,15]. Importantly, while many studies favor LBBAP over RVA pacing for preserving LVEF, some observational data suggest that RVA pacing still yields comparable clinical outcomes in selected populations, particularly older adults or those with significant comorbidities [16]. It is important to note that the risks associated with RVA pacing are not uniform across all patient populations. In patients with preserved ejection fraction, minimal pacing requirements, or limited life expectancy, the clinical impact of RVA pacing may be negligible, and its procedural simplicity makes it an entirely reasonable choice. Therefore, while the overall trend in modern pacing has shifted toward physiological strategies, RVA pacing remains clinically appropriate in well-selected cases—especially where technical limitations, anatomical constraints, or resource availability preclude more advanced approaches.

Given this complex and evolving landscape, the choice between the apex and septum—and, more broadly, between conventional versus conduction system pacing—remains an area of intense clinical debate. Understanding the strengths and limitations of each strategy is essential for optimizing patient outcomes. This review critically examines current evidence comparing these pacing sites, with a focus on their long-term clinical outcomes, procedural challenges, and patient selection considerations. In this context, “implantation strategy” specifically refers to the deliberate choice of pacing site and the corresponding technical approach and tools needed to optimize physiological pacing and patient benefit.

## 2. Right Ventricular Apex Pacing: Historical Standard and Limitations

**Figure 1:**
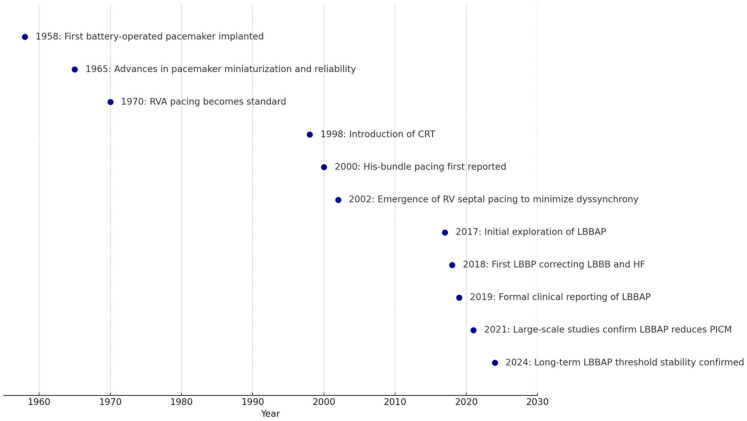
Key milestones in evolution of cardiac pacing strategies. CRT: cardiac resynchronization therapy; LBBB: left bundle branch block; LBBP: left bundle branch pacing; LBBAP: left bundle branch area pacing; PICM: pacemaker-induced cardiomyopathy; RVA: right ventricular apex.

The history of cardiac pacing dates back to 1958, when Dr. Åke Senning and engineer Rune Elmqvist implanted the first battery-operated pacemaker (Figure 1) [1]. The early devices were large, short-lived, and rudimentary compared to today’s sophisticated systems. During the 1960s and 1970s, advancements in technology led to more reliable and miniaturized pacemakers, enabling complete implantation within the body (Figure 1) [1]. As cardiac pacing techniques evolved, the RVA quickly became the standard site for lead placement. This preference was largely driven by the ease of venous access, technical simplicity, and the stable sensing and pacing thresholds associated with apical positioning. During this era, emphasis was placed primarily on achieving consistent electrical capture rather than considering the long-term hemodynamic consequences of artificial pacing. As a result, RVA pacing was widely adopted as the default strategy, and it continues to be a common practice globally.

Despite this, growing evidence—especially from the past two decades—has demonstrated that persistent RVA pacing may exert deleterious effects on ventricular function, ultimately impacting overall patient prognosis. One of the principal complications is the increased risk of developing HF. The Mode Selection Trial (MOST), a landmark randomized controlled trial, showed that patients who experienced more than 40% right ventricular pacing had a 2.6-fold higher risk of HF hospitalization compared to those with lower pacing burdens over a median follow-up of 33.1 months [17,18]. Another key finding of the study was the emergence of de novo HF. As the authors included patients with preserved LV function, the results suggested that chronic high-burden RVA pacing can induce new-onset HF even in structurally normal hearts [17,18]. Existing data suggest that the risk of HF—whether de novo or worsening of pre-existing dysfunction—might be influenced not only by the cumulative percentage of ventricular pacing (“pacing burden”), but also by the degree of pacing-induced mechanical desynchrony.

Beyond HF, chronic RVA pacing has also been implicated in the development of PICM: a recognized clinical entity characterized by a significant decline in left ventricular ejection fraction directly attributable to sustained right ventricular stimulation. A study by Khurshid et al. estimated that approximately 13% of patients with chronic RVA pacing develop PICM [19], with the risk escalating dramatically in those subjected to a higher percentage of ventricular pacing (>40–50%), reaching incidences as high as 20–30% [20].

In addition to its role in HF (development/progression) and PICM, high pacing burden has also been associated with increased all-cause mortality. A sub-analysis of the MOST trial showed that patients with high RV pacing burdens had a 37% increased adjusted risk of mortality (HR 1.37; 95% CI: 1.05–1.79; *p* = 0.02), when compared to those with minimal pacing [18]. A study by Kiehl et al. further reinforced these findings, demonstrating a 6.3% increase in all-cause mortality among patients undergoing chronic high-burden RVA pacing [20]. These findings underscore the critical importance of reducing pacing burden—not only as a strategy to prevent HF, but also to potentially improve long-term survival outcomes. This recognition catalyzed the development of modern pacing algorithms such as Ventricular Intrinsic Preference (VIP™) and Managed Ventricular Pacing (MVP™), which aim to promote intrinsic conduction and minimize unnecessary ventricular pacing. Clinical studies have demonstrated that the VIP™ algorithm reduced unnecessary RV pacing from 97% to 39% at 12 months (*p* = 0.0004), while MVP™ achieved up to a 99% reduction [21,22].

Despite these technological advances, the clinical impacts of pacing minimization strategies on HF outcomes remain nuanced. Trials such as the SAVE PACE study [21] have demonstrated that MVP™ algorithms substantially reduced RV pacing burden but failed to show a significant reduction in HF hospitalizations over a mean follow-up of 1.7 years. Similarly, the MINERVA trial [23] confirmed that minimizing RV pacing while promoting intrinsic conduction did not significantly decrease HF-related events during the trial’s follow-up.

These studies revealed that the impact of pacing on cardiac function extends beyond pacing burden alone. They highlighted that while reducing pacing burden is important, the preservation of physiologic electrical activation is fundamental to preventing pacing-induced cardiac dysfunction. This concept has been further substantiated in a recent meta-analysis of 14 randomized controlled trials involving 885 patients [4], which demonstrated a mean LVEF reduction of 3.35% (95% CI: 1.80–4.91) with chronic high-burden RVA pacing; as well as complementary real-world evidence from a Medicare administrative cohort [24] showing a 1.8-fold increased risk of HF hospitalization in contemporary patients with impaired LVEF, despite optimal utilization of modern programming algorithms.

In addition to the recognized long-term complications of RVA pacing, lead-related mechanical complications represent an important consideration. Although relatively rare, lead perforation can have severe clinical consequences. In a large retrospective cohort study, the authors reported an incidence of approximately 0.8% for cardiac perforation following permanent pacemaker implantation, with RVA lead placement identified as a contributing risk factor (Table 1) [25]. Perforation of the right ventricular free wall may result in pericardial effusion, cardiac tamponade, and, in severe cases, cardiogenic shock requiring emergent intervention. Factors contributing to an increased risk of perforation include advanced patient age, corticosteroid use, active fixation leads, and excessive lead slack or force during deployment. Although the absolute incidence remains low, the potential severity of RVA lead perforation adds further complexity to the risk–benefit assessment of chronic apical pacing, especially when considering long-term outcomes in vulnerable populations.

Despite all that has been said and while substantial evidence highlights the risks of chronic high-burden RVA pacing—particularly in terms of the development of HF, PICM, and increased mortality—these adverse outcomes are not uniformly observed across all patient populations. Indeed, the detrimental effects appear to be most pronounced in patients with impaired baseline ventricular function, high pacing dependency, or long-term exposure to desynchronized activation patterns.

Conversely, in selected cohorts—such as older adults, patients with preserved ejection fraction, or those with minimal expected pacing requirements—RVA pacing has demonstrated acceptable long-term outcomes. Therefore, rather than dismissing RVA pacing outright, its continued use in contemporary practice reflects a rational, individualized approach to device therapy with the aim of balancing patient characteristics, anatomical feasibility, and procedural constraints. It was precisely the recognition of these limitations in certain high-risk populations, however, that drove the evolution of alternative pacing strategies such as CSP in earnest during the early 2000s (Figure 1). Initial efforts focused on modifying the lead position within the right ventricle itself, targeting the interventricular septum or right ventricular outflow tract (RVOT). It was hypothesized that pacing at these sites—which are closer to the natural conduction pathways—would promote a more physiological ventricular activation sequence, minimizing the mechanical desynchrony associated with traditional RVA pacing.

Prior to the emergence of CSP, the introduction of cardiac resynchronization therapy (CRT) in the late 1990s (see Figure 1) revolutionized the management of HF by correcting electrical desynchrony through biventricular pacing [26]. CRT established the principle that resynchronizing ventricular contraction could significantly improve outcomes in HF patients with conduction delays.

Early observational studies and small randomized trials lent support to the concept of non-apical pacing. For instance, the PROTECT-PACE trial demonstrated that patients randomized to RV septal or high septal lead placement exhibited better preservation of LVEF compared to those with apical pacing after 12 months of follow-up [27]. Similarly, the SEPTAL study revealed that pacing the mid-septum was safe, feasible, and associated with shorter QRS durations, suggesting improved electrical synchrony [28]. However, the clinical benefits of septal pacing were somewhat inconsistent across studies, partly due to the inherent difficulty in reliably confirming true septal lead placement with standard fluoroscopic techniques alone.

## 3. Conduction System Pacing: His-Bundle and Left Bundle Branch Area Approaches

In parallel, a new paradigm emerged with the concept of CSP, which aims to restore native ventricular activation rather than simply approximating it. His-bundle pacing, first reported by Deshmukh et al. in 2000 (Figure 1), represented a landmark innovation involving the delivery of electrical stimulation directly to the His–Purkinje network [29]. Early experiences with HBP showed remarkable improvements in electrical synchrony, reflected by narrower paced QRS complexes and preserved left ventricular function; particularly in patients requiring high-burden pacing or with baseline conduction disease. In a prospective observational study involving HF patients with LBBB, HBP successfully corrected LBBB in 97% of cases, leading to significant improvements in LVEF from 32.4 ± 8.9% to 55.9 ± 10.7% (*p* < 0.001) over a mean follow-up of 37 months [30]. Similarly, the HOPE-HF randomized, double-blind, crossover trial demonstrated that HBP improved quality of life and was symptomatically preferred by the majority of patients, without adversely impacting ventricular function during the six-month study period [31].

Despite these advantages, HBP poses considerable technical and practical challenges, with high and unstable pacing thresholds, lead instability, and difficulty in achieving selective His capture having significantly limited its widespread applicability. Long-term follow-up studies provide compelling quantitative evidence of these limitations. In a multicenter study by Watanabe et al., patients with initially higher HBP thresholds (1.25–2.49 V at 1.0 ms) exhibited a 50% incidence of capture thresholds rising above 2.5 V over a mean follow-up of 34.6 months, compared to only 14% in patients with lower initial thresholds (*p* = 0.023) [9]. Additionally, the same study reported a lead revision rate of 21% and HBP abandonment in 29% of the high-threshold group, underscoring the technical instability associated with HBP [9]. Further corroborating these findings, Padala and Ellenbogen observed in a large two-center cohort that mean pacing thresholds increased from 1.6 V at implantation to 2.0 V after three years, with 28% of patients demonstrating thresholds ≥ 2.5 V during follow-up and approximately 35–40% experiencing significant troubleshooting issues, including threshold rise and lead revisions [32].

Approximately 11–15% of intra-procedural lead repositioning in HBP patients is attributed to technical challenges, such as difficulty in achieving selective His capture or unstable electrical parameters [9]. To address this, pre-implant mapping is often required to identify the optimal His-bundle recording site—an approach that extends procedure times and increases fluoroscopy exposure. A large multicenter observational study involving 529 patients across seven international centers reported an overall HBP implantation success rate of 81% [33]. Notably, centers that had completed the initial learning curve of around 40 cases achieved success rates as high as 87%, highlighting the critical role of operator experience in procedural outcomes [34]. Supporting this, the international Physiologic Pacing Registry, which included 849 patients from 44 centers, reported a comparable success rate of 88.5% [35]. However, when stricter criteria for acceptable electrical performance were applied—specifically, a capture threshold of ≤3 V at a pulse width of ≤1.0 ms—the effective success rate declined to 79.8% [34]. Collectively, these findings illustrate that while HBP offers a physiologically ideal pacing approach, its clinical adoption is hampered by sensitivity to threshold elevations and mechanical instability, which have tempered initial optimism.

Driven by the need for a more technically feasible alternative that preserves physiologic activation, LBBAP was introduced as a more feasible and robust form of conduction system engagement [36,37,38]. The earliest conceptualization of LBBAP can be traced to the pioneering work of Huang et al. who, in 2017 (Figure 1), first reported successful left bundle branch pacing (LBBP) in HF patients requiring CRT [38].

Rapidly gaining attention, the LBBP technique evolved from initial attempts to achieve deep septal pacing to more targeted engagement of the left bundle system, with its deeper, more distal septal lead positioning distinguishing it from HBP. Initial investigations demonstrated that LBBP not only provides bradycardia support but can also correct LBBB and restore synchronous ventricular contraction in HF patients, offering a physiologically appealing alternative to conventional CRT (Table 1) [38,39]. Recognizing the variability in achieving pure left bundle capture, the definition of LBBP was broadened to “left bundle branch area pacing (LBBAP),” encompassing direct left bundle capture and adjacent septal pacing, thus enhancing procedural feasibility and clinical adoption.

Early prospective studies—notably those by Jastrzębski et al.—demonstrated that LBBAP achieved high procedural success rates (exceeding 90%) with stable, low pacing thresholds (typically <1.0 V) and excellent electrical synchrony, as reflected by significantly narrowed QRS complexes (mean reduction of ~30–40 ms compared to baseline) [11]; see Table 1.

Compared to HBP, LBBAP offers consistently higher acute implantation success rates, with large multicenter registries and observational studies reporting success rates between 85% and 95% [11,40]. This advantage stems from the broader and more accessible target area within the interventricular septum, which reduces procedural complexity and minimizes reliance on precise anatomical localization.

Complication rates in large contemporary cohorts are also favorable: lead dislodgement has been reported in just 0.3% to 1.6% of cases, and septal perforation remains uncommon [11,40,41,42]. Iatrogenic right bundle branch block (RBBB)—reflected as a paced RBBB morphology on ECG—is frequently observed during successful LBBAP due to early left ventricular activation with relatively delayed right ventricular conduction. Although exact incidence rates are inconsistently reported, this ECG pattern is considered an expected and benign consequence of selective left bundle capture rather than a pathological complication and does not typically require intervention [11,43].

The refinement of implantation techniques—such as sheath angiography and contrast injection—has further enhanced procedural control and minimized deep septal misplacement.

Importantly, longitudinal data have demonstrated that pacing thresholds in LBBAP remain remarkably stable over time, with mean thresholds showing an insignificant increase from 0.8 ± 0.5 V at implant to 0.9 ± 0.5 V at long-term follow-up (*p* = 0.35), in stark contrast to the significant threshold increase observed in HBP (*p* < 0.001) [44]. This stability minimizes concerns regarding threshold increases, thereby preserving device battery longevity and reducing the need for lead revision.

Notably, LBBAP consistently achieves narrower paced QRS durations (averaging 110–130 ms), which is considered as a surrogate marker of improved ventricular synchrony compared to RVA and even HBP in some studies.

LBBAP also shows promising effects on cardiac function, HF, and PICM. A comprehensive meta-analysis comprising 3906 patients with predominantly baseline HF (mean LVEF ~45%) and a mean follow-up of approximately 12–18 months revealed that LBBAP significantly improved LVEF (+4.78%, 95% CI: 3.26–6.30) and reduced HF hospitalization rates (OR: 0.44, 95% CI: 0.29–0.68), when compared to biventricular pacing [45]. Moreover, LBBAP was associated with a remarkable decrease in the incidence of PICM compared to RVA. In a prospective cohort study involving 235 patients, Li et al. reported a PICM incidence of only 2.5%—a stark reduction compared to the 13–30% PICM incidence consistently observed with chronic high burden RVA pacing [46]. Even HBP—although superior to RVA in reducing PICM (5.8% vs. 22% at one year in the His-SYNC trial) [47]—does not achieve the same consistently low incidence seen with LBBAP.

In current practice, LBBAP is increasingly favored in specific clinical scenarios where CRT or HBP may be less feasible or effective. Examples include patients with failed or nonresponding CRT implants, those with narrow QRS complexes who require high-burden pacing but present with challenging His-bundle anatomy, or cases where HBP is limited by persistently high thresholds or lead instability [32,40]. Recent multicenter studies and randomized trials have begun directly comparing LBBAP with HBP and conventional CRT; for example, the MELOS study [11] and the International LBBAP Collaborative [40] demonstrate that LBBAP can achieve comparable or even superior outcomes in terms of electrical synchrony and procedural feasibility in well-selected patients. These emerging data are expected to refine clinical decision-making and further define the optimal role of LBBAP within physiological pacing strategies.

Despite all these demonstrated compelling early outcomes with LBBAP, it is important to interpret the current evidence with appropriate caution. A substantial proportion of the available data stems from observational studies, registries, and retrospective cohorts. These study designs are inherently susceptible to selection bias, lack of blinding, and confounding by indication. For example, patients selected for LBBAP may have more favorable baseline characteristics, and procedural success is often higher in specialized centers with extensive operator experience. Additionally, the promising clinical outcomes published to date may reflect an element of publication bias, with successful case series more likely to be reported than neutral or negative experiences. Notably, only a limited number of randomized controlled trials comparing LBBAP to standard pacing modalities are currently available, and long-term data (>5 years) remain sparse [44,48]. As such, while LBBAP shows great promise, the technique should be viewed as an evolving modality whose definitive role will be clarified through future large-scale, multicenter, randomized trials with long-term clinical and safety endpoints.

Of the few studies providing insights into outcomes approaching or exceeding five years of follow-up, most notably, we can report on a multicenter cohort assessed by Wang et al., who followed 237 patients post transcatheter aortic valve implantation (TAVI) who received either LBBAP or traditional RVA [49]. While the composite outcome of all-cause death did not differ significantly between the groups, the LBBAP cohort showed a notable 65% relative reduction in HF hospitalization at 5 years (7.5% vs. 21.4%; adjusted HR: 2.26; 95% CI: 1.01–5.08; *p* = 0.048). Additionally, LBBAP was associated with a significantly narrower paced QRS duration (122 ± 12 ms vs. 151 ± 18 ms) and better preservation of left ventricular ejection fraction over time (LVEF change: +6% vs. +3%, *p* = 0.046). These findings provide one of the first meaningful indications that the acute hemodynamic advantages of LBBAP may translate into superior long-term clinical outcomes in certain high-risk populations.

### Leadless Pacemakers: Expanding Physiological Pacing Options

Leadless pacemakers represent another important recent advancement in bradycardia device therapy. By eliminating transvenous leads and subcutaneous pockets, these devices significantly reduce the risk of lead-related complications, pocket infections, and venous obstruction, offering a minimally invasive alternative for patients with limited venous access, prior device infections, or high infection risk. Despite these advantages, leadless pacemakers are not universally suitable. Until recently, most commercially available devices provided only single-chamber ventricular pacing, limiting their use to patients who do not require atrial sensing or atrioventricular (AV) synchrony. Ongoing developments—including new-generation dual-chamber and atrioventricular synchronous leadless systems—aim to expand indications and improve physiologic pacing options.

Another practical consideration is device fixation and long-term stability: early designs faced challenges with dislodgement or suboptimal positioning, but modern leadless systems employ improved active fixation mechanisms to enhance anchoring within the myocardium. Importantly, precise placement is still essential. These devices are typically deployed in the mid-interventricular septum to ensure stable pacing thresholds and to avoid interference with the tricuspid valve apparatus. Implantation that is too basal—even if septal—can increase the risk of new or worsening tricuspid regurgitation due to leaflet impingement [50]. Finally, the inability to extract or reposition a leadless device once endothelialized may present future challenges as multiple devices accumulate or battery depletion occurs, although retrieval techniques and battery longevity are actively improving.

As leadless pacing technology continues to mature, further large-scale studies and real-world experience will help clarify patient selection, procedural refinements, and its role alongside CSP and CRT within modern individualized pacing strategies.

## 4. Conclusions

Despite the widespread shift toward physiologic pacing, the debate between RVA pacing and LBBAP remains far from settled. While mounting evidence supports LBBAP as a strategy that better preserves left ventricular function and reduces PICM—particularly in patients with high pacing burdens—it is not without limitations. Technical challenges such as achieving consistent conduction system capture, navigating septal fibrosis, and the risk of septal perforation persist, often requiring significant operator experience, imaging support, and institutional infrastructure.

Conversely, RVA pacing, though historically associated with deleterious effects on cardiac function in some cohorts, continues to demonstrate acceptable clinical outcomes in specific populations. In patients with preserved ejection fraction, minimal pacing requirements, or limited life expectancy, studies suggest that RVA pacing remains a safe and pragmatic choice. Its procedural simplicity, broad availability, and consistently high implantation success rates make it particularly valuable in settings where anatomical complexity or resource limitations preclude more advanced techniques.

Therefore, the choice between RVA and LBBAP should not be viewed through a binary lens of superiority but, rather, as a matter of individualized clinical judgment. The selection of an optimal pacing strategy requires the consideration of patient-specific factors such as underlying cardiac function, anticipated pacing burden, anatomical feasibility, operator expertise, and institutional capabilities. As the field of cardiac pacing continues to evolve, future research—particularly large-scale, randomized trials—will be essential in defining clear indications and refining best practices.

Finally, despite LBBAP’s higher costs due to specialized instrumentation and technical complexity, its potential to reduce PICM, heart failure hospitalizations, and lead revisions may offer long-term economic value for carefully selected patients. Further robust health-economic studies will be essential to validate these assumptions and guide optimal resource use.

Until then, the question of “apex or septum” remains not only clinically relevant but also central to the broader pursuit of tailored, evidence-based care in modern electrophysiology.

## Figures and Tables

**Table 1:** Comparative evaluation of right ventricular apex pacing, His-bundle pacing, and left bundle branch area pacing across procedural and clinical outcomes.

Parameter	RVA Pacing	HBP	LBBAP
Implantation success rate	≥95%	81–88.5%	85–95%
Lead dislodgement Rate (post-implant)	1–2%	~3–5%	0.3–1.6%
Septal/RV perforation rate	0.8–1%	<1%	0.5–1%
Induction of RBBB	No	Rare	Common
Change in LVEF	Decline (−3.35%)	Reserved/mild improvement	Improvement (+4.78%)
Risk of new-onset HF	Increased (1.8%)	Reduced (vs. RVA)	Significantly reduced (OR 0.44)
Incidence of PICM	12–20%	5.8%	2.5–3%
Technical complexity	Low	High	Moderate
Need for mapping tools	No	Yes	Minimal
Pacing threshold stability	Stable	Threshold often increased	Stable

HBP: His-bundle pacing; HF: heart failure; LBBAP: left bundle branch area pacing; LVEF: left ventricular ejection fraction; OR: odds ratio; PICM: pacing-induced cardiomyopathy; RBBB: right bundle branch block; RV: right ventricle, RVA: right ventricular apex.

## Data Availability

Not applicable.
